# Impact of Natriuretic Peptide on the Evolution of Patients With Pulmonary Embolism and Neoplasm

**DOI:** 10.7759/cureus.73853

**Published:** 2024-11-17

**Authors:** Daniela Maria Nemtut, Ruxandra Ulmeanu, Noémi Németh, Cristina Tudoran, Alexandru Motofelea, Florica Voita-Mekeres, Lavinia Davidescu

**Affiliations:** 1 Cardiology, Pelican Hospital, Oradea, ROU; 2 Pulmonology, North Hospital, Provita Medical Group, Bucharest, ROU; 3 Doctoral Studies Department, Biomedical Science, University of Oradea, Oradea, ROU; 4 Cardiology, Victor Babeş University of Medicine and Pharmacy, Timisoara, ROU; 5 Internal Medicine, Victor Babeş University of Medicine and Pharmacy, Timisoara, ROU; 6 Psychiatry, University of Oradea, Oradea, ROU; 7 Pulmonology, Faculty of Medicine and Pharmacy, University of Oradea, Oradea, ROU; 8 Pulmonology, Hospital of Pneumology, Oradea, ROU

**Keywords:** early mortality, hemo-dynamic status, late mortality, nt-probnp, oncological pathology, prognosis, recurrence, risk of complications, risk stratification

## Abstract

This retrospective study investigated the prognostic value of N-terminal pro-brain natriuretic peptide (NT-proBNP) in 106 patients with pulmonary embolism (PE) and associated oncological pathology. The study aimed to evaluate the predictive accuracy of NT-proBNP for both the prognosis and complication risk, such as early mortality (≤ 30 days), late mortality (≥ 30 days), and PE recurrence, in relation to the neoplasm's location and stage. Additionally, it explored the relationship between NT-proBNP, hemodynamic status (stable/unstable), and the location of PE in the pulmonary arteries (main, lobar, segmental) for prognostic and complication risk assessment. The results showed that cancer patients with NT-proBNP levels above 600 ng/L had a significantly higher risk of acute PE recurrence compared to those with lower levels, especially in cases involving the main pulmonary arteries. Hemodynamic instability further elevated the risk of PE recurrence and death, underscoring the importance of NT-proBNP as a prognostic marker for this population. Patients with unstable hemodynamic status were more likely to have elevated NT-proBNP levels, and this was associated with a markedly increased risk of early as well as late demise. Furthermore, patients with multiple tumor locations demonstrated a heightened risk of mortality when NT-proBNP levels were elevated. These findings highlight the potential of NT-proBNP as a valuable tool for risk stratification and patient management in individuals with PE and associated oncological pathology.

## Introduction

Pulmonary embolism (PE) is a critical medical condition characterized by a significant early mortality rate. It ranks as the third most common cause of cardiovascular death following acute myocardial infarction and stroke. Pulmonary artery occlusion can be caused by thrombi or other emboli, such as tumors, fat, or air [1,2]. Approximately one-quarter of patients experience sudden death; in survivors, it can lead to the development of chronic thromboembolic pulmonary hypertension and recurrent PE over time. Mortality risk, both initially and long-term, depends on the severity of the acute manifestation, recurrence of PE, and associated conditions. The highest risk of developing PE is within the first 12 months following a cancer diagnosis, mainly due to treatments like chemotherapy and surgery [3,4]. This risk progressively declines, so much so that 10 years after a cancer diagnosis, the risk of thromboembolic events approximates that of the general population. PE can often be the first presentation of an underlying malignancy or arise as a complication of a known malignancy.

The reactivity of cardiac biomarkers such as N-terminal pro-B-type natriuretic peptide (NT-proBNP) and troponins in patients with PE and neoplasms is attributed to right ventricular dysfunction, often due to right ventricular dilation secondary to a sudden increase in pulmonary artery pressure [5]. Biomarkers such as NT-proBNP, d-dimer, myoglobin, and troponin correlate with right ventricular dysfunction [6]. In acute PE, elevated concentrations of NT-proBNP and troponin are associated with increased mortality risk, while normal serum NT-proBNP levels suggest a favorable prognosis [7,8].

Natriuretic peptides are sensitive indicators of right ventricular dysfunction in PE cases [9,10]. Right ventricular dysfunction, commonly seen in acute PE, is often detected by echocardiography or computed tomography. It is characterized by an increased right-to-left ventricular ratio and may be linked to electrocardiographic abnormalities and elevated NT-proBNP, leading to a higher mortality [11]. Normal NT-proBNP levels in hemodynamically stable PE patients correlate with lower mortality risk, whereas elevated NT-proBNP and troponin levels, markers of cardiac injury, are associated with increased early in-hospital mortality. However, neither biomarker directly guides clinical treatment decisions for hemodynamically stable patients [9].

Although elevated troponin and myoglobin levels suggest myocardial damage, no conclusive evidence indicates they are superior to NT-proBNP levels as severity indicators. NT-proBNP is now considered the strongest predictor of PE severity, as shown by a 2008 study by N. Vuilleumier. Only NT-proBNP showed a correlation with right ventricular dysfunction on chest CT, a key negative prognostic indicator for PE. Nevertheless, NT-proBNP is not a definitive indicator for thrombolysis [9].

NT-proBNP and troponin 1 levels, markers of atrial and myocardial stress due to overload, are poor prognostic indicators in acute PE [12]. NT-proBNP is produced as an inactive prohormone (proBNP) that cleaves into the active brain natriuretic peptide (BNP) hormone and the inactive N-terminal fragment (NT-proBNP) [13]. Natriuretic peptides are essential diagnostic and prognostic biomarkers for congestive heart failure. BNP is mainly produced by ventricular myocytes, in contrast to atrial natriuretic peptide, which is primarily synthesized in atrial tissue. BNP levels rise in response to myocardial stretch [14].

The clinical significance of BNP lies in its capacity to diagnose, treat, and predict adverse outcomes in heart failure cases. BNP is released in response to increased intracardiac volume and pressure, with myocardial stretching as the primary trigger, and it is regulated by catecholamines and angiotensin II [15,16]. BNP, the active peptide, inhibits the renin-angiotensin-aldosterone system (RAAS) and the sympathetic nervous system (SNS), relaxes vascular smooth muscle cells, and prevents renal tubular sodium reabsorption, leading to vasodilation, natriuresis, diuresis, reduced blood volume, and lower blood pressure [15,17]. Elevated BNP levels due to ventricular stress are poor prognostic indicators in PE cases. High NT-proBNP levels are seen in various conditions, including left ventricular dysfunction, advanced age, renal failure, and chronic pulmonary disease [18].

Though BNP and NT-proBNP are generally linked to myocardial stress, increasing evidence suggests a positive relationship between NT-proBNP and inflammatory cytokines like interleukin-6 (IL-6). In cancer patients, elevated BNP levels can occur without clinical heart failure, correlating with high-sensitivity C-reactive protein (hs-CRP) and reflecting cancer-associated inflammation. BNP levels can rise significantly in severe sepsis and septic shock [19-21].

High levels of natriuretic peptides are observed in right ventricular pressure overload conditions, including primary pulmonary hypertension, chronic thromboembolic pulmonary hypertension, and chronic lung disease [22,23].

This study aimed to evaluate the predictive accuracy of NT-proBNP as a cardiac biomarker for prognosis and risk of complications, such as early mortality (≤ 30 days), late mortality (> 30 days), and recurrence of PE, in relation to the location and stage of associated neoplasms. It also explored the correlation between NT-proBNP and hemodynamic stability (stable/unstable) to enhance prognostic predictions and reduce complications in this patient group. Additionally, this study examined NT-proBNP levels across specific sites of PE within the main, lobar, and segmental pulmonary arteries to assess prognosis and the complication risk based on the embolism's anatomical location.

## Materials and methods

This retrospective study included 106 patients hospitalized and treated in the Cardiology Department of Oradea County Emergency Clinical Hospital and the Oncology Department of "Dr. Gavril Curteanu" Municipal Hospital Oradea between January 1, 2019, and December 31, 2021. All included subjects had a PE and associated oncological pathology, with different localizations and clinical stages. This study was approved by the Ethics Committee of Oradea County Emergency Clinical Hospital on December 12, 2019, with registration number 30278/12.12.2019, and by the Ethics Committee of "Dr. Gavril Curteanu" Municipal Hospital Oradea on November 5, 2020, with registration number 31465/11/5/2020.

The cohort sample consisted of individuals who were Romanian citizens. Data collection was conducted in a way that guaranteed patient anonymity. The study included patients who met the following criteria: patients diagnosed with acute PE, according to the 2019 European Society of Cardiology (ESC) guidelines and having associated oncological pathology, regardless of the location, staging, or treatment (surgical, chemotherapy, radiotherapy) of the neoplasm. All included patients were over 18 years of age. Patients with recurrent PE and associated oncological pathology were also included.

Statistical analysis

Continuous variables were tested for normality using the Shapiro-Wilk test. Normally distributed data were reported as means±standard deviations (SD), while non-normally distributed data were presented as medians with interquartile ranges (IQRs; 25th to 75th percentiles). Categorical variables were described using counts and percentages. Group differences for normally distributed continuous variables were analyzed with Welch's t-test (for two groups) and one-way ANOVA (for multiple groups) with post-hoc tests (e.g., Tukey's HSD). For non-parametric continuous data, the Mann-Whitney U and Kruskal-Wallis tests were used for two and multiple groups, respectively, followed by Dunn's post-hoc tests as needed. Comparisons of categorical data utilized Chi-square tests or Fisher's exact test when expected cell counts were below five. Binomial logistic regression identified independent predictors of study outcomes, accounting for potential confounders. Multicollinearity among predictors was checked before any analysis in order to maintain model integrity. Logistic regression coefficients quantified the association between independent and outcome variables, with significance determined by p-values less than 0.05. Model performance and suitability were evaluated using a pseudo-R-squared measure and the Hosmer-Lemeshow goodness-of-fit test. Sample size calculations were performed a priori to achieve a 95% confidence level and 80% statistical power based on anticipated effect sizes and variance from preliminary data. All statistical analyses were conducted in R (version 3.6.3), using various packages within the Tidyverse for data manipulation and visualization, final fit for regression analyses, and other specialized packages (MCGV, Stringdist, Janitor, Hmisc) for specific data processing needs. Biomarker levels (NT-proBNP) were quantified using the Pathfast Compact Immuno-Analyzer in whole blood.

## Results

Our study involved 106 patients. 45.28% (48 individuals) were male, diagnosed with PE and active oncological pathology, exhibiting various localizations and clinical stages. Patients were categorized according to the occurrence of reacted or non-reacted NTproBNP, state of hemodynamic stability (stable/unstable), stage and location of the neoplasm, and location of the thrombus in the pulmonary arteries (main, lobar, segmental, subsegmental).

The mean age of the patients was 69.2 years, with a standard deviation of 12.6 and an age range of 29 to 94. The gender distribution was relatively balanced, with a slightly higher proportion of females 58 (54.7%) compared to males 48 (45.3%). Regarding the residential environment, more patients came from urban areas 60 (56.6%) compared to rural areas 46 (43.4%), although this difference was not statistically significant (p=0.053).

A significant proportion of the patients, 66 individuals (62.3%), had NTproBNP levels below 600 ng/L, while 40 patients (37.7%) had NTproBNP values above 600 ng/L. In terms of cancer staging, the majority of patients, 46 (58.2%), had T4 stage cancer, with a decreasing distribution among lower stages: 21 (26.6%) were classified as T3, 9 (11.4%) as T2, and 3 (3.8%) as T1. Notably, 27 patients were unstaged.

As for cancer location, the digestive system was the most common site, affecting 31 patients (29.2%). Lung cancer followed, involving 28 patients (26.4%). Urogenital system cancers were found in 26 patients (24.5%), while breast cancer was observed in eight patients (7.5%). Lymphomas and other locations made up smaller portions of the population at 5.7% and 6.6%, respectively (Table 1).

**Table 1:** Demographic and clinical characteristics of the study population

	Overall (N=106)	Percentage
Age		
Mean (SD)	69.2 (12.6)	
Range	29.0 - 94.0	
Gender		
Female	58	54.7%
Male	48	45.3%
Area		
Rural	46	43.4%
Urban	60	56.6%
NTproBNP		
<600 ng/L	66	62.3%
>600 ng/L	40	37.7%
Stage		
Unstaged	27	
T1	3	3.8%
T2	9	11.4%
T3	21	26.6%
T4	46	58.2%
Location of cancer		
Breast	8	7.5%
Digestive system	31	29.2%
Lung	28	26.4%
Lymphomas	6	5.7%
Other location	7	6.6%
Urogenital system	26	24.5%

Our study included 27 patients, of whom PE was the initial manifestation of cancer. The initial diagnosis of PE was documented in 35.85% of cases, followed by PE post-chemotherapy (26.42%) and PE post-surgery (18.87%).

Our study revealed that cancer was most frequently located in the digestive system with 31 patients (29.2%) and in the lungs with 28 patients (26.4%). A total of 26 patients (24.5%) had cancer in the urogenital system, while 8 (7.5%) had breast cancer, and 6 (5.7%) had lymphomas. Patients with urogenital neoplasms had the highest incidence of NT-proBNP levels above 600 ng/L, with 12 individuals (30.0%), although this was not significantly higher than patients with other localizations, such as the digestive system (13 patients, 32.5%, p=0.3771), lung (10 patients, 25.0%, p=0.3771), breast (2 patients, 5.0%, p=0.3771), or other locations (3 patients, 7.5%, p=0.3771). Interestingly, none of the lymphoma patients had NT-proBNP levels above 600 ng/L (Table 2).

**Table 2:** Distribution of cancer location by NT-proBNP levels in the study population (N=106) 1. Pearson's Chi-squared test NT-proBNP: N-terminal pro-brain natriuretic peptide

	NT proBNP <600 ng/L (N=66)	Percentages	NT-proBNP >600 ng/L (N=40)	Percentages	Total (N=106)	Percentages	p-value
Cancer location							0.377^1^
Breast	6.0	9.1%	2.0	5.0%	8.0	7.5%	
Digestive system	18.0	27.3%	13.0	32.5%	31.0	29.2%	
Lung	18.0	27.3%	10.0	25.0%	28.0	26.4%	
Lymphomas	6.0	9.1%	0.0 (0.0%)		6.0	5.7%	
Other location	4.0	6.1%	3.0	7.5%	7.0	6.6%	
Urogenital system	14.0	21.2%)	12.0	30.0%	26.0	24.5%	

NTproBNP and Pulmonary Embolism Recurrences

In oncologic patients, the recurrence risk was 4.2 times higher in patients with NTproBNP above 600 ng/L compared to those with NTproBNP below 600 ng/L (RR=4.242, 95% CI: 0.5417-33.2258, z=1.376, p=0.169).

No significant difference in mortality was observed between stage T2 patients with NTproBNP < 600 ng/L compared to those with NTproBNP > 600 ng/L in both early and late deaths (p=0.2531 and p=0.5951, respectively). Similarly, stage T1 patients with NTproBNP > 600 ng/L did not exhibit a significantly higher mortality risk compared to those with NTproBNP < 600 ng/L for both early (p=0.4331) and late deaths (p=0.2631).

For stage T3, while early mortality was slightly higher in patients with NTproBNP < 600 ng/L (25.0%) compared to NTproBNP > 600 ng/L (14.3%), this difference was not statistically significant (p=0.5811). Late mortality also showed no significant difference between the two groups (p=0.1721).

In stage T4, late mortality was higher in patients with NTproBNP > 600 ng/L (57.6%) compared to those with NTproBNP < 600 ng/L (37.0%), approaching statistical significance (p=0.0621). However, early mortality in stage T4 patients did not significantly differ between those with NTproBNP > 600 ng/L and <600 ng/L (p=0.1611) (Table 3).

**Table 3:** Comparison of early and late deaths in relation to tumor staging and serum marker levels 1. Pearson’s Chi-squared test was used to determine statistical significance for both early deaths (p-value^1^) and late deaths (p-value^2^)

Stage		<600 ng/L Early deaths, N=12	Percentages	>600 ng/L Early deaths, N=7	Percentages	Total Early deaths, N=19	Percentages	p-value^1^	<600 ng/L Late deaths, N=54	Percentages	>600 ng/L Late deaths, N=33	Percentages	Total Late deaths, N=87	Percentages	p-value^2^
T1	No	11.0	91.7%	7.0	100.0%	18.0	94.7%	0.4331	52.0	96.3%	33.0	100.0%	85.0	97.7%	0.2631
	Yes	1.0	8.3%	0.0	0.0%	1.0	5.3%		2.0	3.7%	0.0	0.0%	2.0	2.3%	
T2	No	10.0	83.3%	7.0	100.0%	17.0	89.5%	0.2531	49.0	90.7%	31.0	93.9%	80.0	92.0%	0.5951
	Yes	2.0	16.7%	0.0	0.0%	2.0	10.5%		5.0	9.3%	2.0	6.1%	7.0	8.0%	
T3	No	9.0	75.0%	6.0	85.7%	15.0	78.9%	0.5811	41.0	75.9%	29.0	87.9%	70.0	80.5%	0.1721
	Yes	3.0	25.0%	1.0	14.3%	4.0	21.1%		13.0	24.1%	4.0	12.1%	17.0	19.5%	
T4	No	9.0	75.0%	3.0	42.9%	12.0	63.2%	0.1611	34.0	63.0%	14.0	42.4%	48.0	55.2%	0.0621
	Yes	3.0	25.0%	4.0	57.1%	7.0	36.8%		20.0	37.0%	19.0	57.6%	39.0	44.8%	

NT-proBNP levels above 600 ng/L were predicted based on the location of the cancer. The intercept of the model was 0.405, corresponding to an odds ratio of 1.5 (95% CI: 0.2506-8.98), with a p-value of 0.657, indicating no significant effect at baseline.

For breast cancer patients, the odds of having NT-proBNP levels above 600 ng/L were lower compared to the reference group ("Other location"), with an estimated log odds of -1.322. This corresponds to an odds ratio of 0.267 (95% CI: 0.0235-3.02), though the difference was not statistically significant (p = 0.286).

Similarly, for patients with digestive system cancer, the estimated log odds were -0.742, yielding an odds ratio of 0.476 (95% CI: 0.0668-3.40), with a p-value of 0.459. This suggests that digestive system cancer patients are less likely to have NT-proBNP levels above 600 ng/L compared to those with cancers in other locations, though this result was also not statistically significant.

Lung cancer patients exhibited a log odds of -0.405, translating to an odds ratio of 0.667 (95% CI: 0.0803-5.54), with a p-value of 0.707, indicating no significant association between lung cancer and elevated NT-proBNP levels relative to the reference group.

For lymphoma patients, the model produced an extreme log-odds estimate of -16.972, which corresponds to an odds ratio near zero (4.26e-8) and an extremely wide confidence interval (95% CI: 0.0000-Inf). This result, with a p-value of 0.992, suggests no reliable or significant relationship between lymphoma and NT-proBNP levels above 600 ng/L.

Finally, for patients with urogenital system cancers, the log odds were -0.272, with an odds ratio of 0.762 (95% CI: 0.0974-5.96) and a p-value of 0.796, indicating no significant difference when compared to the reference group (Table 4).

**Table 4:** Logistic regression model predicting NT-proBNP levels (>600 ng/L) based on cancer location Note: Estimates represent the log odds of NT-proBNP levels being "> 600 ng/L" versus "< 600 ng/L." Inf: Indicates an infinite value in the upper confidence interval bound, arising when the probability of an outcome is near zero, leading to an unbounded upper interval. Estimate: The estimated coefficient for each predictor, representing the log odds of NT-proBNP levels > 600 ng/L relative to those < 600 ng/L. SE (Standard Error): Measures the precision of the estimate; NT-proBNP: N-terminal pro-brain natriuretic peptide Z: The Z-value, calculated as the Estimate divided by SE, used in hypothesis testing. Odds Ratio: The exponential of the estimate, showing the odds of NT-proBNP > 600 ng/L relative to the reference category. 95% CI Lower/Upper: The lower and upper bounds of the 95% confidence interval for the odds ratio, providing a range within which the true odds ratio is expected to fall.

Predictor	Estimate	SE	z-value	p-value	Odds Ratio	95% CI Lower	95% CI Upper
Intercept	0.405	0.913	0.4442	0.657	1.5	0.2506	8.98
Cancer location:							
Breast – Other location	-1.322	1.238	1.0674	0.286	0.267	0.0235	3.02
Digestive system – Other location	-0.742	1.002	0.7402	0.459	0.476	0.0668	3.4
Lung – Other location	-0.405	1.08	0.3754	0.707	0.667	0.0803	5.54
Lymphomas – Other location	-16.972	1696.735	0.01	0.992	4.26E-08	0	Inf
Urogenital system – Other location	-0.272	1.049	0.2591	0.796	0.762	0.0974	5.96

Evaluation of the Relationship Between NTproBNP and Hemodynamic Status (Stable/Unstable) for Prognosis and Risk of Complications in This Category of Patients

Among patients with early deaths, NT-proBNP levels below 600 ng/L were observed in 88.9% (32 out of 36) of patients who had no hemodynamic instability, while 11.1% (4 out of 36) experienced hemodynamic instability. In contrast, for those with NT-proBNP levels above 600 ng/L, 75.9% (22 out of 29) had no hemodynamic instability, and 24.1% (7 out of 29) experienced hemodynamic instability. The difference in hemodynamic instability between the two groups (NT-proBNP <600 ng/L vs. NT-proBNP >600 ng/L) was not statistically significant (p = 0.1641).

For patients with late deaths, NT-proBNP levels below 600 ng/L were seen in 86.7% (26 out of 30) of patients with no hemodynamic instability, while 13.3% (4 out of 30) experienced hemodynamic instability. In contrast, for those with NT-proBNP levels above 600 ng/L, only 45.5% (5 out of 11) had no hemodynamic instability, and 54.5% (6 out of 11) experienced hemodynamic instability. The difference in hemodynamic instability between the two groups (NT-proBNP <600 ng/L vs. NT-proBNP >600 ng/L) was statistically significant (p = 0.0061) (Table 5).

NTproBNP levels above 600 ng/L were detected in 59.09% of oncologic patients with unstable hemodynamic status, which was not significantly different from those with NTproBNP below 600 ng/L (40.91%, p=0.233).

**Table 5:** Comparison of early and late deaths based on hemodynamic instability and NTproBNP Levels 1. Pearson Chi-Square NT-proBNP: N-terminal pro-brain natriuretic peptide

	Early Deaths							Late Deaths					
	<600 ng/L (N=36)	Percentages	>600 ng/L (N=29)		Total (N=65)		p-value	<600 ng/L (N=30)		>600 ng/L (N=11)		Total (N=41)	p-value
Hemodynamic instability							0.16411						0.00611
No	32	88.90%	22	75.90%	54	83.10%		26	86.70%	5	45.50%	31	75.60%
Yes	4	11.10%	7	24.10%	11	16.90%		4	13.30%	6	54.50%	10	24.40%

NTproBNP levels above 600 ng/L were detected in 59.09% of oncologic patients with unstable hemodynamic status, which was not significantly different from those with NTproBNP below 600 ng/L (40.91%, p=0.233).

For those with stable hemodynamic status, NTproBNP levels greater than 600 ng/L were present in only 32.14% of cases, significantly lower than those with NTproBNP less than 600 ng/L (67.87%, p<0.001).

Compared to patients with stable hemodynamic status, the prevalence of NTproBNP levels above 600 ng/L was significantly higher in patients with unstable status (59.09% vs 32.14%, p=0.021).

In the group of patients with unstable hemodynamic status, the likelihood of having NTproBNP levels above 600 ng/L was 1.8 times greater in patients with unstable hemodynamic status than in those with stable status (RR=1.838, 95%CI: 1.533-2.931, z=2.559, p=0.011).

Oncologic patients with elevated NTproBNP levels and unstable hemodynamic status had a 6.6-fold increased risk of recurrence compared to those with stable status (RR=6.600, 95%CI: 0.251-173.621, z=1.131, p=0.258). On the other hand, patients with NTproBNP<600 ng/L had no risk of recurrence independent of their hemodynamic status (RR=1,063, 95%CI: 0.113-10.025, z=0.053, p=0.958).

There was no significant difference in mortality rates between oncologic patients with NTproBNP > 600 ng/L, 38.46% and those with stable status (62.96%, p=0.150), both for early (15.38% vs 18.52%, p=0.809) and late deaths (23.08% vs 44.44%, p=0.197).

Within the group of oncologic patients with NTproBNP < 600 ng/L, 44.44% of those with unstable hemodynamic status experienced death. This percentage was not significantly different from those with stable status (59.65%, p=0.395). Although the rate of early deaths was greater in the stable group (0.00% vs 21.05%, p=0.131), the difference was not significant. Higher rates of late deaths were observed in the unstable group (44.44% vs 38.60%, p=0.741).

The risk of mortality in oncologic patients with unstable hemodynamic status was 1.2 times higher for those with NTproBNP levels above 600 ng/L compared to those with NTproBNP levels below 600 ng/L (RR=1.156, 95% CI: 0.424-3.151, z=0.282, p=0.778).

The risk of early death in oncologic patients with unstable hemodynamic status was 3.6 times higher for those with NTproBNP levels above 600 ng/L (RR=3.571, 95%CI: 0.192-66.615, z=0.853, p=0.394).

Evaluation of the Relationship Between NTproBNP and the Location of the PE in the Main Pulmonary Arteries, Lobar, and Segmental Arteries for Prognosis and Risk of Complications in This Category of Patients

PE in the main pulmonary artery occurred in 24.2% (16 out of 66). For those with NT-proBNP levels above 600 ng/L, 80.0% (32 out of 40) did not have a PE in the main pulmonary artery, while 20.0% (8 out of 40) had a PE. The difference in the occurrence of PE in the main pulmonary artery between the two groups was not statistically significant (p = 0.613).

In the group with NT-proBNP levels below 600 ng/L, 63.6% (42 out of 66) had a PE in the lobar artery, while 36.4% (24 out of 66) did not have a PE. For those with NT-proBNP levels above 600 ng/L, 72.5% (29 out of 40) had a PE in the lobar artery, while 27.5% (11 out of 40) had no PE. The difference in the occurrence of PE in the lobar artery between the two groups was not statistically significant (p = 0.347).

Among patients with NT-proBNP levels below 600 ng/L, 12.1% (8 out of 66) had a PE in the segmental or subsegmental artery, while 87.9% (58 out of 66) had no PE. For those with NT-proBNP levels above 600 ng/L, 7.5% (3 out of 40) had a PE in the segmental or subsegmental artery, while 92.5% (37 out of 40) had no PE. The difference in the occurrence of PE in the segmental/subsegmental artery between the two groups was not statistically significant (p = 0.450).

Most PEs were located in the lobar arteries (66.98%), while 22.64% were observed in the main pulmonary arteries. In oncologic patients, the highest proportion of NT-proBNP > 600 ng/L was found in those patients with a PE localized in the main pulmonary arteries (45.83%). However, this percentage was not significantly greater than those with PE localized in the lobar pulmonary arteries or segmental pulmonary arteries (32.21%, p=0.356, and 36.36%, p=0.509, respectively) (Table 6).

**Table 6:** Comparison of pulmonary embolism locations based on NT-proBNP levels in the study population NT-proBNP: N-terminal pro-brain natriuretic peptide; PE: Pulmonary embolism

	<600 ng/L (N=66)	Percentages	>600 ng/L (N=40)	Percentages	Total (N=106)	Percentages	p-value
PE: Main pulmonary artery massive							0.613
No	50.0	75.8%	32.0	80.0%	82.0	77.4%	
Yes	16.0	24.2%	8.0	20.0%	24.0	22.6%	
PE: Lobar artery							0.347
No	24.0	36.4%	11.0	27.5%	35.0	33.0%	
Yes	42.0	63.6%	29.0	72.5%	71.0	67.0%	
PE: Segmental/subsegmental artery							0.450
No	58.0	87.9%	37.0	92.5%	95.0	89.6%	
Yes	8.0	12.1%	3.0	7.5%	11.0	10.4%	

Among patients with early deaths and NT-proBNP levels below 600 ng/L, 86.7% (26 out of 30) did not have a PE in the main pulmonary artery, while 13.3% (4 out of 30) had a PE. For those with NT-proBNP levels above 600 ng/L, 90.9% (10 out of 11) did not have a PE in the main pulmonary artery, while 9.1% (1 out of 11) had a PE. The difference in the occurrence of PE in the main pulmonary artery between the two groups was not statistically significant (p = 0.7131).

In the group with late deaths and NT-proBNP levels below 600 ng/L, 66.7% (24 out of 36) did not have a PE in the main pulmonary artery, while 33.3% (12 out of 36) had a PE. For those with NT-proBNP levels above 600 ng/L, 75.9% (22 out of 29) did not have a PE in the main pulmonary artery, while 24.1% (7 out of 29) had a PE. The difference between the two groups was not statistically significant (p = 0.4181).

Among patients with early deaths and NT-proBNP levels below 600 ng/L, 73.3% (22 out of 30) had a PE in the lobar artery, while 26.7% (8 out of 30) did not have a PE. For those with NT-proBNP levels above 600 ng/L, 81.8% (9 out of 11) had a PE in the lobar artery, while 18.2% (2 out of 11) did not have a PE. The difference in PE occurrence in the lobar artery between the two groups was not statistically significant (p = 0.5751).

In the group with late deaths and NT-proBNP levels below 600 ng/L, 55.6% (20 out of 36) had a PE in the lobar artery, while 44.4% (16 out of 36) did not have a PE. For those with NT-proBNP levels above 600 ng/L, 69.0% (20 out of 29) had a PE in the lobar artery, while 31.0% (9 out of 29) did not have a PE. The difference between the two groups was not statistically significant (p = 0.2691).

Among patients with early deaths and NT-proBNP levels below 600 ng/L, 86.7% (26 out of 30) did not have a PE in the segmental or subsegmental arteries, while 13.3% (4 out of 30) did have a PE. For those with NT-proBNP levels above 600 ng/L, 90.9% (10 out of 11) did not have a PE in these arteries, while 9.1% (1 out of 11) did have a PE. The difference in the occurrence of PE in the segmental/subsegmental arteries between the two groups was not statistically significant (p = 0.7131).

In the group with late deaths and NT-proBNP levels below 600 ng/L, 88.9% (32 out of 36) did not have a PE in the segmental or subsegmental arteries, while 11.1% (4 out of 36) did have a PE. For those with NT-proBNP levels above 600 ng/L, 93.1% (27 out of 29) did not have a PE in these arteries, while 6.9% (2 out of 29) did have a PE. The difference between the two groups was not statistically significant (p = 0.5601) (Table 7).

**Table 7:** Comparison of early and late deaths based on pulmonary embolism locations and NT-proBNP levels NT-proBNP: N-terminal pro-brain natriuretic peptide; PE: Pulmonary embolism

PE Location	<600 ng/L (Early deaths, N=30)	Percentages	>600 ng/L (Early deaths, N=11)	Percentages	Total (Early deaths, N=41)	Percentages	p-value (Early deaths)	<600 ng/L (Late deaths, N=36)	Percentages	>600 ng/L (Late deaths, N=29)	Percentages	Total (Late deaths, N=65)	Percentages	p-value (Late deaths)
Main pulmonary artery							0.7131							0.4181
No	26.0	86.7%	10.0	90.9%	36.0	87.8%		24.0	66.7%	22.0	75.9%	46.0	70.8%	
Yes	4.0	13.3%	1.0	9.1%	5.0	12.2%		12.0	33.3%	7.0	24.1%	19.0	29.2%	
Lobar artery							0.5751							0.2691
No	8.0	26.7%	2.0	18.2%	10.0	24.4%		16.0	44.4%	9.0	31.0%	25.0	38.5%	
Yes	22.0	73.3%	9.0	81.8%	31.0	75.6%		20.0	55.6%	20.0	69.0%	40.0	61.5%	
Segmental and subsegmental arteries							0.7131							0.5601
No	26.0	86.7%	10.0	90.9%	36.0	87.8%		32.0	88.9%	27.0	93.1%	59.0	90.8%	
Yes	4.0	13.3%	1.0	9.1%	5.0	12.2%		4.0	11.1%	2.0	6.9%	6.0	9.2%	

## Discussion

The marker NT-proBNP can be regarded as an indicator of short-term mortality. Further studies are required to establish the potential involvement of NT-proBNP measurement levels in determining thrombolysis treatment and in identifying patients suitable for outpatient treatment [12,24]. NT-proBNP levels in patients with PE are advised due to their status as a prognostic biomarker [25-27].

Several meta-analyses have examined the prognostic value of measuring natriuretic peptides in patients with PE and have consistently found that increased levels of natriuretic peptides are linked to a negative short-term prognosis [25].

A threshold value of 600 ng/L for NTproBNP is used to stratify the risk of death in hemodynamically stable patients with PE [26]. Patients with NT-proBNP levels above 600 ng/L, right ventricular systolic dysfunction, and increased PESI score have a poor prognosis [27]. Patients exhibiting a PE and NT-proBNP < 600 ng/L, normal right ventricular function, and a PESI score of 0 have been linked to a favorable prognosis [26].

An investigation carried out by Lega et al. [26] involving 688 patients found that patients with a PE, stable hemodynamics, right ventricular systolic dysfunction, and NT-proBNP > 600 ng/L may benefit from early thrombolytic therapy. 

Elevated serum levels of NT-proBNP > 600 ng/L are linked to high mortality in PE, whereas low serum levels of NT-proBNP < 600 ng/L are predictive of a favorable prognosis [28]. 

Although increased NT-proBNP levels indicate patients with a higher risk of complications or death, they do not warrant the initiation of invasive therapies. Conversely, normal NT-proBNP levels can help identify patients who may benefit from outpatient treatment. In a 2008 study conducted by Frederikus A. Klok, patients with increased serum levels of NT-proBNP had a 45% frequency of right ventricular dysfunction compared to patients with normal serum levels of NT-proBNP, who had a 4.5% incidence of right ventricular dysfunction [12]. Our study emphasizes the ability of NT-proBNP levels to serve as a risk stratification tool for predicting hemodynamic instability and mortality in patients with PE and associated oncologic pathology. The results indicate that patients who have higher serum levels of NT-proBNP, exceeding 600 ng/L, are more susceptible to hemodynamic instability and face a poorer prognosis and a greater risk for both short-term and long-term complications.

Therefore, NT-proBNP can serve as a valuable tool in identifying patients at high risk of hemodynamic instability who may benefit from improved monitoring or alternative therapeutic approaches to decrease mortality in these patients. Current limitations exist in the capacity to predict immediate and delayed complications. Research has established NT-proBNP is the most accurate indicator of mortality [29,30]. These biomarkers can serve as reliable indicators for predicting mortality and morbidity in PE.

NT-proBNP is an easily accessible and cost-effective biomarker that can be measured at the time of a PE diagnosis to enhance prognosis, prevent complications, and lower risks in both the acute and long-term phases in patients with PE and neoplasms [31,32].

The limitations of our study included the absence of long-term patient follow-up, lack of dosage of troponins, and the study being carried out in a single center with a relatively small patient population. To achieve statistical significance, future research should confirm our results at a multicenter level and in a larger patient population.

Our analysis revealed that NT-proBNP serves as a prognostic marker. Specifically, patients with unstable status had NT-proBNP values > 600 ng/l, significantly higher than those with stable hemodynamic status (59.09% vs 32.14%, p = 0.021). Similarly, among cancer patients, NT-proBNP values > 600 ng/L were 1.8 times greater in patients with hemodynamic instability compared to those with stable status.

The current study found that cancer patients with unstable status have a 1.2-fold increased risk of death when their NT-proBNP values are above 600 ng/L compared to those with NT-proBNP values below 600 ng/L. The risk of early death in oncologic patients with unstable status was 3.6 times higher when NT-proBNP values exceeded 600 ng/L. The mortality risk was 2.7 times higher for those with NT-proBNP > 600 ng/L compared to those with NT-proBNP < 600 ng/L. Thus, the risk of early death is 1.3 times greater for this population, while the risk of late mortality is 6.8 times higher. In cancer patients with unstable status and NT-proBNP > 600 ng/L, the mortality risk is 9.2 times higher compared to non-oncologic patients.

## Conclusions

Our study revealed that cancer patients with NT-proBNP levels above 600 ng/L have a significantly higher risk of acute PE recurrence compared to those with lower levels, especially in cases involving the main pulmonary arteries. Hemodynamic instability further elevated the risk of PE recurrence and death, underscoring the importance of NT-proBNP as a prognostic marker in this population.

Patients with unstable hemodynamic status were more likely to have elevated NTproBNP levels, and this was associated with a markedly increased risk of both early and late deaths. Additionally, patients with multiple tumor locations demonstrated a heightened risk of mortality when NT-proBNP levels were elevated.

Interestingly, the mortality rate for early deaths did not significantly differ between patients with higher and lower NT-proBNP levels when a PE was located in the lobar pulmonary arteries, indicating that the location of the embolism plays a role in outcomes.
